# Primary Fallopian Tube Carcinoma Arising in the Setting of Chronic Pelvic Inflammatory Disease

**DOI:** 10.1155/2014/645045

**Published:** 2014-01-12

**Authors:** Ibrahim M. Zardawi

**Affiliations:** Pathology Department, Port Macquarie Base Hospital, Port Macquarie, NSW 2444, Australia

## Abstract

Primary fallopian tube cancer (PFTC) is a rare gynaecological malignancy, clinically often mistaken for pelvic inflammatory disease or ovarian cancer. Three primary fallopian tube carcinomas, arising in a background of chronic pelvic inflammatory disease (PID), are presented. The possible association between chronic PID and PFTC is discussed and a hypothesies linking these cancers with chronic inflammation is proposed.

## 1. Introduction

Primary fallopian tube cancer (PFTC) is a relatively aggressive gynaecological malignancy. It accounts for 1% of all female genital malignancies [1–3]. The aetiology of PFTC is unknown but nulliparous women and women with germ-line BRCA mutation and women in certain occupations such as smelting workers, artistic workers, hairdressers, packers, nurses, shop workers, and clerical workers appear to be at increased risk of PFTC [4–7]. Serous papillary carcinoma is the most common histological type, accounting for >90% of the cases with these tumours. PID is very common and *Chlamydia trachomatis* infection is one of the most significant causes of chronic uterine tube inflammation [8]. Chlamydial infection has been associated with certain cancers [7, 9, 10]. This paper describes 3 cases of PFTC, clinically presenting as tuboovarian abscesses. A possible link between chronic PID and PFTC is suggested.

## 2. Materials and Methods

Three cases of primary fallopian tube carcinomas, presenting between April 2011 and August 2013 with a clinical impression of an inflammatory tuboovarian mass, are described.

**Figure 1:**
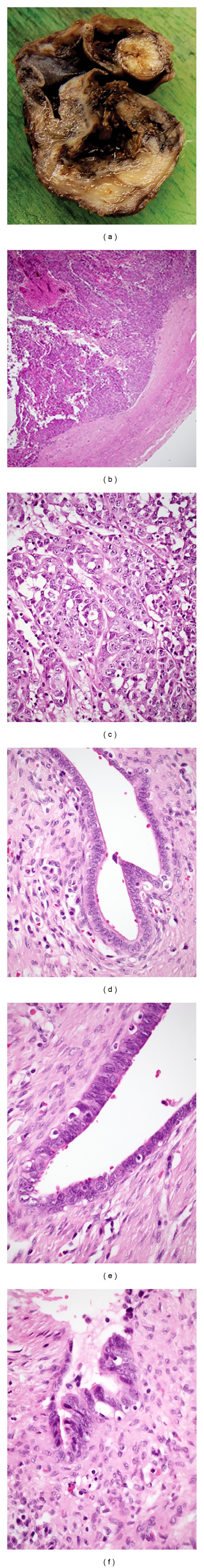
(a) is the macroscopic image of the left fallopian tube which is totally destroyed and cystic. Fleshy white masses are noted in the wall. The upper nodular mass protrudes into the lumen of the cystically dilated haemorrhagic tube. (b) is a low power view of the tube with the tumour protruding into the lumen (Haematoxylin and Eosin stained section, original magnification of ×4). (c) is high power view of the tumour (Haematoxylin and Eosin stained section, original magnification of ×60). (d) shows the benign tubal epithelium (Haematoxylin and Eosin stained section, original magnification of ×60) and (e) and (f) show dysplastic tubal epithelium with loss of polarity, stratification, and nuclear pleomorphism (Haematoxylin and Eosin stained sections, original magnification of ×60).

Case 1 is a 46-year-old Indigenous woman, admitted in April 2011 with pelvic pain. Abdominal ultrasound showed a 130 mm complex left cystic adnexal mass which was clinically considered to be a tuboovarian abscess as there was a longstanding history of PID due to *Chlamydia* and gonorrhoea. No free fluid was seen in the pelvis and a CA-125 was normal. Abdominal computerised tomography confirmed the adnexal lesion but showed no additional masses. She had recently been investigated for menorrhagia and her endometrial biopsy and cervical cytology had been normal. She has type 2 diabetes and hypertension. On vaginal examination a large left sided pelvic mass was felt. At laparotomy there were dense adhesions between the adnexae, uterus, and bowel. The adnexae were completely obscured by scar tissues. She underwent hysterectomy left salpingo-oophorectomy and omental biopsy. Macroscopically, the left adnexa (65 × 60 mm) hands a globular tan brown cystic appearance with a thickened fleshy wall and multiple fleshy projections into the lumen (Figure 1). A high grade mainly solid serous carcinoma was seen in the tube microscopically (Figure 1). Although the fallopian tube was almost completely destroyed by scar tissue from past PID, close relationship to uninvolved and “premalignant” dysplastic tubal epithelia could be microscopically established (Figure 1). The ovary was not identified. The tumour appeared to be limited to the tube and was staged as TNM stage pT1A (FIGO Stage IA). The tubal cancer was regarded as a primary fallopian tubal carcinoma, for which the patient received 6 cycles of postoperative adjuvant therapy. Posttreatment restaging CT 6 weeks later was normal and serial serum CA-125 measurements remained normal. The patient is alive and apparently disease free.

**Figure 2:**
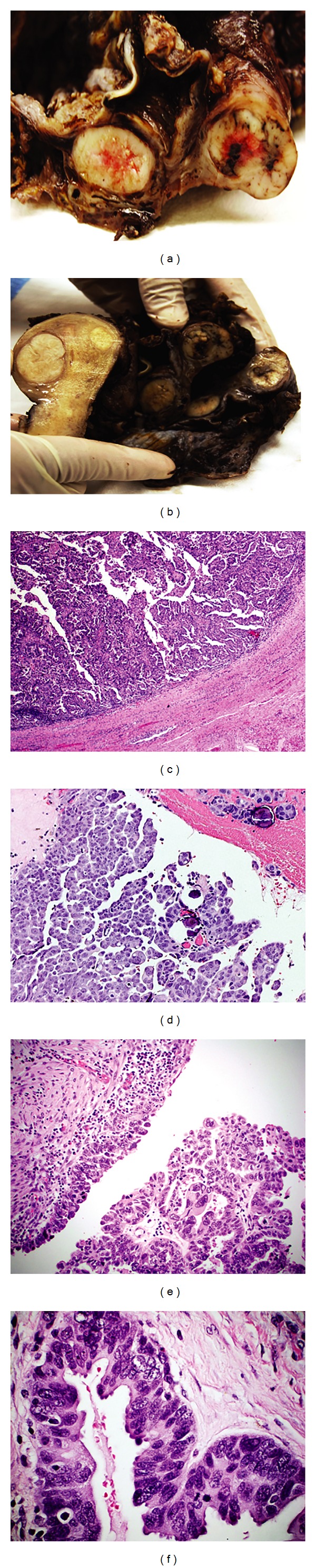
(a) is the macroscopic image of the left fallopian tube which shows a cross section of sausage-like white fleshy mass within what appears to be part of the tube. The remainder of the tube is destroyed and cystic. (b) shows the right tube and uterus. The right tube is somewhat similar to the left and shows 2 tumour masses. The uterus with simple leiomyomas is also shown. (c) is a panel that is a low power view of the left tube with a papillary carcinoma (Haematoxylin and Eosin stained section, original magnification of ×10) and (d) is high power view of the right tube with a serous carcinoma, displaying a micropapillary growth pattern with occasional psammoma bodies (Haematoxylin and Eosin stained section, original magnification of ×40). (e) shows a higher power view of the left tubal tumour (right side) and high grade dysplasia in lining epithelium on the left side (Haematoxylin and Eosin stained section, original magnification of ×60) and (f) shows high grade dysplastic epithelium in the right tube (Haematoxylin and Eosin stained section, original magnification of ×60).

Case 2 is a 50-year-old Indigenous woman who presented with diarrhoeal illness, right abdominal pain, poorly controlled type 2 diabetes mellitus, and abdominal sepsis in May 2012. She also complained of intermenstrual bleeding and had previously been treated for PID due to *Chlamydia*. Hysteroscopy and endometrial biopsy in 2010 revealed no pathology and her cervical cytology was also normal. CT abdomen and pelvis showed bilateral adnexal cysts with the right measuring 120 mm and the left measuring 102 mm. Fluid-filled tubal structures were noted in close proximity of the cystic lesions on both sides. Some of the right-sided cysts communicated directly with the fallopian tube. There was also a heterogeneous thick-walled cystic lesion in the vicinity of the right-sided fluid-filled tube. These were interpreted as inflammatory collections. CA-125 was markedly elevated at 1900 kU/L (reference range < 35 kU/L). She underwent total abdominal hysterectomy and bilateral salpingo-oophorectomy. In view of her clinical status and the presence of adhesions, temporary large bowel enterostomy and peritoneal washings were also performed. Macroscopic examination showed a uterus, cervix, tubes, and ovaries, 266 gm. The uterine corpus contained multiple leiomyomas but the cervix appeared unremarkable. The left (175 × 90 × 60 mm) and right (190 × 80 × 60 mm) adnexae were distorted by cystic lesions. Both tubes also had multiple tan and brown polypoid luminal areas (Figure 2). Histologically, a high grade serous cystadenocarcinoma, originating from the right fallopian tube and involving both adnexal regions and greater omentum (Figure 2), was identified. PID was also identified. No normal ovarian tissue was recognised. The cervix, endometrium, and myometrium showed no malignancy. The peritoneal washings revealed inflammatory material only. The tumour was staged as INM pT3aNXMX (FIGO Stage IIIA). She received chemotherapy and is being followed up with no evidence of disease recurrence clinically or by CA-125 levels.

**Figure 3:**
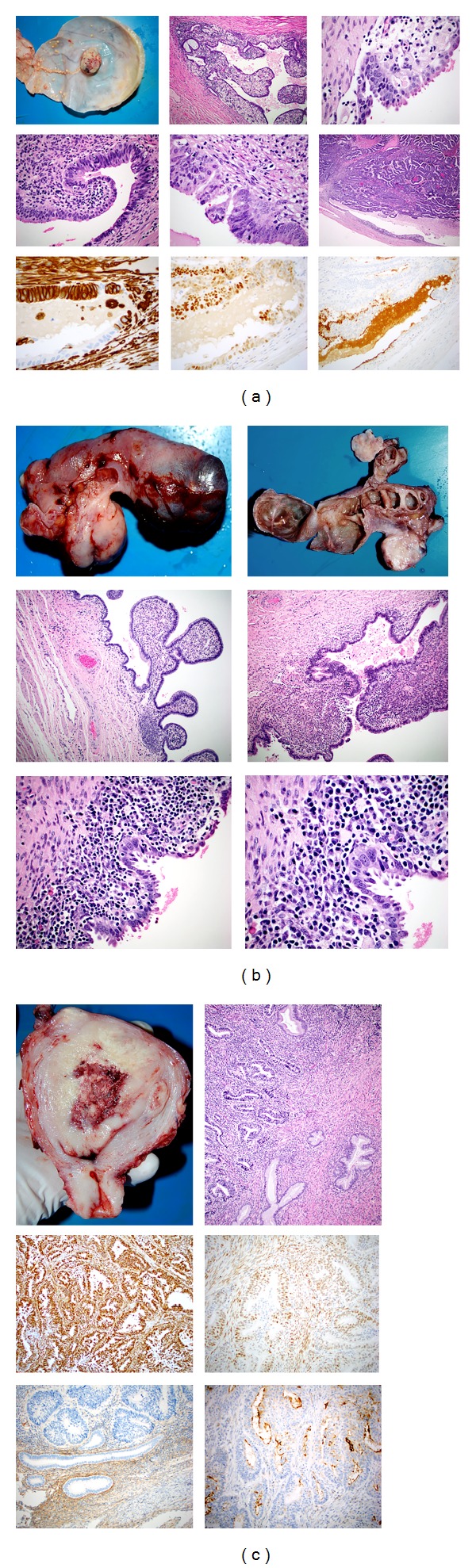
(a) The upper left panel is the macroscopic image of the right fallopian tube which is markedly distended and shows a polypoid mucosal mass (right tube). Small yellowish deposits are also seen in the mucosa. The upper middle and right panels show chronic xanthomatous salpingitis with lymphoid and foam cell infiltration. The middle panel is the Haematoxylin and Eosin stained section with an original magnification of ×10 and the right panel which is the also Haematoxylin and Eosin stained section with an original magnification of ×40. The left panel of the middle row shows tubal epithelium near the polypoid lesion with high grade dysplasia (Haematoxylin and Eosin stained section, original magnification ×40). The middle panel of the middle row also shows tubal epithelium near the polypoid lesion with high grade dysplasia (Haematoxylin and Eosin stained section, original magnification of ×60). The right panel of the middle row shows the polypoid mass (Haematoxylin and Eosin stained section, original magnification of ×4). The left panel of the lower row is a vimentin stain which stains the stroma and the dysplastic epithelium but not the normal tubal epithelium (immunoperoxidase stain, original magnification ×60). The middle panel of the lower row is immunoperoxidase stain for oestrogen receptor with an original magnification ×40. The dysplastic tubal epithelium is strongly positive (upper part) and normal tubal epithelium is weakly positive (lower part). The right panel of the lower row is immunoperoxidase stain for CA125 with an original magnification of ×20. The epithelium (normal and dysplastic) shows luminal staining. (b) The upper panels are macroscopic images showing a normal ovary but a distended fallopian tube (left tube). The external surface is shown in the left panel and cut surface is shown in the right panel. The tube shows multiple fibrous bands. The middle panels show features of chronic salpingitis with disorganisation of tubal mucosal folds, infiltration by inflammatory cells, fibrosis, and loss of muscle. The left panel of the middle row is the Haematoxylin and Eosin stained section with an original magnification of ×4 and the right panel of the middle row is also the Haematoxylin and Eosin stained section but at original magnification of ×10. The lower panels also show features of chronic salpingitis with focal epithelial atypia in the centre of the images. The left panel of the middle row is the Haematoxylin and Eosin stained section with an original magnification of ×40 and the right panel of the middle row is also the Haematoxylin and Eosin stained section but at original magnification of ×60. (c) The left upper panels are macroscopic images of the uterus with a somewhat polypoid endometrial lesion (uterus). The right shows an endometrioid carcinoma which is infiltrating the endocervix (Haematoxylin and Eosin stained section, original magnification of ×4). The left panel of the middle row is a vimentin stain which shows strong stromal and epithelial expression (immunoperoxidase stain, original magnification of ×10). The right panel of the middle row is immunoperoxidase stain for oestrogen receptor with an original magnification of ×20. Positive staining is observed in the stroma as well as the epithelium. The left panel of the lower row is CD10 which stains the normal endometrium (lower part) but not the carcinoma (immunoperoxidase stain, original magnification of ×20). The right panel of the lower row is CA125 which shows luminal staining in the malignant glands (immunoperoxidase stain, original magnification of ×20).

Case 3 is a 43-year-old Indigenous woman who presented in August 2012 with severe menorrhagia, lower abdominal pain, and haemoglobin of 36 g/L. She had a history of chronic PID due to *Chlamydia*. Ultrasound examination confirmed bilateral hydrosalpinx and also a large uterine mass. Her CA-125, CA-19.9, and CEA were normal. Total abdominal hysterectomy, bilateral salpingo-oophorectomy, and peritoneal washings were performed. Macroscopically, the uterus contained a somewhat polypoid endometrial lesion 62 mm and several small leiomyomas up to 15 mm (Figure 3(c)). The right adnexa, which weighed 252 g and measured 130 × 80 × 60 mm, consisted of a cystic mass filled with creamy material. The cystic tubal wall ranged in thickness from 1 mm to 4 mm. A polypoid yellowish tan lesion 20 × 15 mm was seen on the inner surface of the right tube, Figure 3(a). The left adnexa consisted of tuboovarian tissue 60 × 20 mm with a recognisible ovary 20 × 15 × 10 mm. The tube was dilated and filled with haemorrhagic fluid. Multiple septae ranging from 5 mm to 25 mm in length were seen inside the left tube (Figure 3(b)). The uterine lesion was a FIGO grade 3 endometrioid carcinoma. The tumour had extended into the outer myometrium but was clear by 2.5 mm. The endocervix was also involved by carcinoma (Figure 3(c)). The tumour expressed vimentin and oestrogen receptor (Figure 3(c)). CD10 was expressed only in the stroma of the normal endometrium (Figure 3(c)) and CA126 along the luminal border of the malignant glands (Figure 3(a)). The right tube showed xanthomatous salpingitis (Figure 3(a)). The mucosal nodule was a high grade endometrioid carcinoma (Figure 3(a)) which had a similar immunohistochemical profile to the uterine tumour in being oestrogen receptor positive. Vimentin was also positive. CD10 was non contributory and CA125 was expressed along the luminal surface of the malignant glands. In addition, the right tubal epithelial lining also showed high grade dysplasia (Figure 3(a)). The dysplastic epithelium also expressed vimentin, oestrogen receptor, and luminal surface CA125. Both tubes also showed features of hydrosalpynx. The inflammatory process in the left tube was nonspecific (Figure 3(b)) in comparison to the xanthomatous inflammation in the right tube, as previously mentioned (Figure 3(a)). Close examination of the epithelial lining of the left tube showed focal atypia (Figure 3(b)). The was no malignancy in the left tube. The right tubal cancer was limited to the tube without penetration of the serosal surface (TNM stage pT1/FIGO stage IA). Indeed the tubal tumour did not show any mural invasion (Figure 3(a)). In view of the fact that the endometrioid cancer was at a higher stage, the patient was treated as having a TNM stage pT2a/Figo stage IIA. Her follow-up CT of chest, abdomen, and pelvis were unremarkable. The serum CA125 measurement has been normal since surgery.

## 3. Discussion

PFTC accounts for 1% of all gynaecological malignancies. In order to be considered a primary neoplasm, the tumour must be located within the fallopian tube or its fimbriated end and the uterus and ovary must either not contain carcinoma or if they do it must be clearly different from the fallopian tube lesion [11]. The 3 cases presented here fulfill these criteria and are therefore considered primary fallopian tube cancers. The third case is complicated by the fact that a clinically significant primary tumour of the endometrium was also found and the operation was performed for the uterine complaints with the fallopian tube pathology being an incidental finding. The tubal tumour was considered a second primary as it formed a polypoid mucosal lesion (Figure 3(a) left upper panel) with precancerous changes in the mucosa at the base of the polyp (Figure 3(a) middle panels). The tumour was associated with xanthomatous salpingitis (Figure 3(a) middle and right upper panels). The tumour and the precancerous change were endometrioid expressing vimentin and oestrogen receptor (left and middle lower panels). The nonneoplastic tubal tissue did express oestrogen receptor weakly but was not vimentin positive (left and middle lower panels). CA125 was seen only along the luminal surface aspect of the normal, preneoplastic, and neoplastic epithelia (Lower right panel). The endometrial carcinoma showed a similar immunohistochemical profile (Figure 3(c)). The left tube (Figure 3(b)) was not involved by cancer but foci of atypia were seen in the lining mucosa (Figure 3(b) lower panels). The tube, however, did show features of hydrosalpinx (Figure 3(b) upper panels) with chronic salpingitis (Figure 3(b) middle and lower panels). The tumour in the first 2 cases was of the serous type (Figures 1 and 2) and in each case, precancerous changes could be demonstrated in the tube (Figures 1 and 2 lower panels).

Mounting evidence indicates that the vast majority of epithelial ovarian carcinomas are not ovarian in origin. Extrauterine Müllerian epithelium from various sites in the reproductive tract accounts for the diverse morphology and behaviour of these tumours [12]. Prophylactic salpingo-oophorectomies in BRCA positive women have recently implicated the fimbria as a site of origin for high-grade serous carcinoma and its intraepithelial precursors [13–15]. Kurman et al. has proposed a model for the development of ovarian and extraovarian low-grade serous proliferations. They postulate that all of these lesions are derived from papillary tubal hyperplasia, which appears to be induced by chronic inflammation [16]. Chronic inflammation has been implicated in the pathogenesis of a number of human cancers. The mechanisms underlying the relationship between inflammation and cancer are complex. The aetiology of the inflammation is varied and includes microbial, chemical, and physical agents. The chronically inflamed environment which is rich in inflammatory cytokines appears to lead to the activation of a number of signalling pathways [17]. The role of chronic inflammation in the pathogenesis of cancer of the proximal stomach, liver, and uterine cervix with helicobacter pylori, hepatitis B and C, and human papilloma viruses, respectively, is well documented. An association between chronic inflammation and fallopian tube cancer has been suggested by a number of investigators. Lin and colleagues found an association between PID and ovarian cancer and suggested that PID might be a useful marker for ovarian cancer as early detection and treatment could help to improve prognosis. They raised the possibility that pelvic inflammation might accelerate the growth of ovarian cancers or affect cancer-cell differentiation in ways that adversely alter prognosis [18]. Rasmussen et al. found that a history of PID was associated with an increased risk of ovarian borderline tumours and suggested that inflammation could be an etiological factor in borderline tumours [19] and Skapinyecz et al. also found that patients suffering from PID had a higher risk of cervical cancer [20].

Sexually transmitted infections such as chlamydial and gonorrhoeal infections can damage the tubal mucosa. A 60 kDa heat shock protein is believed to be an important triggering pathogenic immune response in chlamydial infection and several studies have demonstrated a correlation between the level of immune response to this chlamydial protein and the extent of tubal damage [21]. *Chlamydia trachomatis* infection is one of the most significant causes of PID. The pathogen is highly prevalent in the cervix and uterine tubes of sexually active women. The probability of having ovarian cancer has been shown to be 90% greater in women with the highest levels of antibodies to chlamydia compared to women with the lowest levels, suggesting that past or chronic persistent chlamydial infection may be a risk factor for ovarian cancer [22]. This possible association was recently revived by J. P. Carvalho and F. M. Carvalho who hypothesised that chlamydial trachomatis infection might be involved in fimbrial carcinogenesis. Fimbrial intraepithelial precursors can evolve into high grade serous carcinomas that spread rapidly to the ovarian surface and peritoneum; such tumours may appear to be primary ovarian neoplasms, though in reality, they are secondary malignancies. J. P. Carvalho and F. M. Carvalho did not offer intracellular signalling pathways for their hypothesis. This hypothesis may have implications for patient management as salpingectomy instead of oophorectomy which may be more appropriate surgery for high risk women [21].

The 3 patients presented here had long standing histories of PIC with many hospital admissions for septic conditions and menstrual irregularities. Chlamydia with or without other infections is usually present in pelvic inflammatory disease and the 3 patients had been previously treated for chlamydial infections. Chlamydia is the most frequently reported notifiable condition in Australia. In 2011 there were almost 80,000 new notifications for persons aged 15 years and over 435 cases per 100,000 population and 9% of chlamydia notifications were among Aboriginal and Torres Strait Islander people, despite this population representing just 2.5% of the total population [8]. The notification rate for the Aboriginal and Torres Strait Islander population was nearly four times that of the non-Indigenous notification rate: 1,257 per 100,000, compared with 340 per 100,000, respectively. Similar to the non-Indigenous population, around 80% of chlamydia diagnoses were among those aged 15 to 29 years. In 2010, more than a third (36%) of all gonorrhoea diagnoses were among Aboriginal and Torres Strait Islander people. The rate of diagnosis was more than 26 times that for the non-Indigenous population: 804 per 100,000, compared with 30 per 100,000, respectively [8]. The 3 patients presented here are from the northern territory of Australia which has a population of approximately 230,000 with 32% being Indigenous [23]. PID is a significant health problem in this cohort and the finding of 3 cases of PFTC is significant and raises the possibility of an association between these cancers and PID.

The role of chlamydial infection is firmly established in conjunctival cancers including marginal zone lymphoma [24, 25]. As chronic chlamydial infection is the most common causes of chronic uterine tube inflammation, this infection is likely to play a role in the pathogenesis of tubal cancer. It is reasonable to suggest that chronic *C. trachomatis* infection may be involved in fimbrial carcinogenesis and subsequent serous carcinomas with the potential for rapid spread to the ovarian surface and peritoneum. This has implications for possible early detection and prophylactic salpingectomy instead of oophorectomy in high risk women.
